# Lipoprotein(a) as a predictor of mortality in hospitalised patients with ischaemic heart disease

**DOI:** 10.3389/fendo.2025.1541712

**Published:** 2025-07-22

**Authors:** Wann Jia Loh, Xuan Han Koh, Colin Yeo, Xucong Ruan, Siang Chew Chai, Weien Chow, Tar Choon Aw, Chew Kiat Heng, Roger Foo

**Affiliations:** ^1^ Department of Endocrinology, Changi General Hospital, Singapore, Singapore; ^2^ Duke-National University Singapore (Duke-NUS) Medical School, Singapore, Singapore; ^3^ Health Services Research, Changi General Hospital, Singapore, Singapore; ^4^ Department of Cardiology, Changi General Hospital, Singapore, Singapore; ^5^ Department of Pathology, Changi General Hospital, Singapore, Singapore; ^6^ Department of Paediatrics, Yong Loo Lin School of Medicine, National University of Singapore, Singapore, Singapore; ^7^ Khoo Teck Puat - National University Children’s Medical Institute, National University Health System, Singapore, Singapore; ^8^ Cardiovascular Translational Research Programme, National University of Singapore, Singapore, Singapore; ^9^ Cardiovascular Research Institute, National University Health System, Singapore, Singapore; ^10^ Genome Institute of Singapore, Agency for Science, Technology and Research, Singapore, Singapore

**Keywords:** lipoprotein(a), Lp(a), coronary artery disease, ischaemic heart disease, Asian, ethnicity, cardiovascular, atherosclerosis

## Abstract

**Background:**

Elevated Lipoprotein(a) [Lp(a)] increases the risk of cardiovascular disease and mortality in population studies but reports of whether elevated Lp(a) concentration predicts mortality in hospitalised patients with cardiovascular disease are still lacking and conflicting.

**Aim:**

To investigate whether elevated Lp(a) predicted cardiovascular outcomes in patients with ischaemic heart disease (IHD) admitted to hospital.

**Methods:**

Serum Lp(a) concentrations were measured in 520 consecutively recruited patients admitted to hospital with IHD, half of whom had an acute myocardial infarction. Patients with elevated Lp(a) at baseline were compared with patients with non-elevated Lp(a). In this observational prospective cohort study, multivariable Cox proportional hazards regression was used to assess the association of baseline Lp(a) with hazard rates (HR) of mortality and major adverse cardiovascular events (MACE).

**Results:**

During the 2-year follow-up period, 14.6%, 8.5%, and 49.2% of patients had all-cause mortality, cardiovascular mortality, and MACE respectively. Median age was 63.5 years, 82.3% were male and the median Lp(a) was 35.2 nmol/L. Multivariable Cox regression showed baseline Lp(a) ≥70 nmol/L was associated with increased risk of all-cause mortality (HR 1.97 [1.20-3.22], *p*=0.007) and cardiovascular mortality (HR 2.01 [1.06-3.82], *p*=0.033), but was not statistically significant for MACE (HR 1.29 [0.98-1.7], *p*=0.067). Higher natural log-transformed Lp(a) concentrations predicted all-cause mortality (HR 1.25 [1.01-1.58], *p*=0.042) but not for cardiovascular mortality or MACE.

**Conclusion:**

In a multi-ethnic Asian patient cohort, elevated Lp(a) concentrations ≥70 nmol/L at hospitalization positively predicted cardiovascular and all-cause mortality in patients with ischaemic heart disease. Our findings support guidelines’ recommendation for routine evaluation of Lp(a) in all patients at high cardiovascular risk.

## 1 Introduction

Elevated lipoprotein(a) blood concentration has consistently shown in multiple large observational and Mendelian randomisation studies to be an independent risk factor for atherosclerotic cardiovascular disease (ASCVD), including ischaemic heart disease (IHD), myocardial infarction, calcific aortic valve stenosis, heart failure, stroke, and cardiovascular mortality (1). Consequently, cardiovascular and lipid guidelines now recommend measuring Lp(a) in all individuals at high risk for atherosclerotic cardiovascular disease (ASCVD), or at least once in all adults during their lifetime (1–4). The majority of these strong evidence comes from prospective population studies, some of which included genetic association analyses (1, 5–9). A meta-analysis in 2022 of 75 studies found that higher Lp(a) concentrations was associated with increased risk of all-cause mortality and CVD mortality for general population and in patients with CVD (9). However, not all studies have consistently shown that Lp(a) is a risk factor for CVD mortality. The LURIC study have reported that Lp(a) concentration in German patients with IHD was not associated all-cause or cardiovascular mortality at a median follow-up of 10 years (10). In a subgroup analysis using NHANES data, the authors reported that Lp(a) was associated with all-cause and CVD mortality for age above 60 years and have BMI >30 kg/m^2^ but not those with age below 60 years or BMI <30 kg/m^2^ (8).

Moreover, the reports are particularly conflicting regarding the prognostic value of Lp(a) in patients admitted to hospital for acute coronary syndromes (ACS) (9–13); An Austrian study of 1245 patients who underwent coronary angiography for ACS, reported that Lp(a) levels at time of ACS did not predict all-cause or cardiovascular mortality, at a median follow up of 5 years (12). Similarly, A Swiss study of 1171 patients hospitalised for ACS reported that Lp(a) at time of angiography was not associated with increased risk of cardiovascular events and mortality at 1 year (13). Nevertheless, a recently published meta-analysis of studies of patients with ACS found that elevated Lp(a) was an independent risk factor for cardiovascular events and all-cause mortality (11). The meta-analysis included Asian data from China, Korea, Japan but there were minimal data from South-east Asia (only a small study from Vietnam) (11). This reflects a pressing issue of the major paucity of data on Lp(a) in South-east Asia. The lack of data in this region is of concern, given the region’s elevated cardiometabolic risk based on ethnicity (14). As Lp(a) distribution and prevalence are ethnicity-dependent, it is also worth noting that prevalence of elevated Lp(a) >50 mg/dL (≈125 nmol/l) varies significantly across different ethnicities from 3 to 30% (15, 16). It is generally accepted that ~20% of the world’s general population has elevated Lp(a) (1). Therefore, the population attributable fraction of Lp(a) to ASCVD risk likely varies across ethnicities, with different cost-economic implications for clinical practice.

Here, we aimed to investigate the mortality risk of elevated Lp(a) in a multi-ethnic Asian cohort of patients at very high-risk of cardiovascular events, focussing on patients who were admitted to hospital with IHD. The primary outcome was mortality risk (all-cause and cardiovascular), and secondary outcome was major adverse cardiovascular events (MACE).

## 2 Methods

### 2.1 Study population

This study was approved by SingHealth Centralised Institution Review Board (CIRB 2020/2065). Our hospital is a large tertiary hospital with 1,000-bed located in eastern Singapore serving more than a million people (20% of the population). Consenting patients with IHD admitted to cardiology wards of Changi General Hospital from June to December 2020 were recruited into our prospective study, underwent medical interviews, blood tests, and observational follow up, as previously described (17, 18). Eligibility for recruitment were based on electronic medical records with a diagnosis of IHD or if there was presence of coronary artery disease. ischaemic cardiomyopathy, coronary artery disease requiring coronary stent intervention or coronary artery bypass graft. A total of 520 patients had Lp(a) measured and completed medical information for analysis in this study. Exclusion criteria for patient recruitment included lack of mental capacity and critically or terminally ill.

### 2.2 Data collection

From the electronic medical records and follow-up phone interviews, data of cardiovascular outcomes for all-cause and cardiovascular-related deaths, and all cardiovascular readmissions were collected. Elective readmissions for procedures were excluded as cardiovascular readmission events. From the electronic medical records and interviews, patient demographics, blood lipid profile, medications, comorbidities were recorded. Lp(a) concentrations were defined as increased when ≥70 nmol/L (≈30 mg/dL), a threshold reported by large observational and epidemiological studies (1).

### 2.3 Outcome assessment

Acute myocardial infarction (AMI) included ST elevation myocardial infarction (STEMI) and non-STEMI. Cardiovascular mortality was defined as death from AMI, IHD, ischemic stroke, end stage heart failure, or other cardiovascular-related causes. A pre-specified composite MACE index was defined as a combination of all-cause mortality, CV mortality and cardiovascular hospital readmissions. Cardiovascular readmissions included hospitalisation for heart failure, stroke, AMI, and other symptomatic cardiac conditions. The medical diagnosis of unstable angina is associated with diagnostic ambiguity and false positives in the clinical setting, hence we did not adopt this terminology (19, 20). We did not have access to the cause of death on the death certificates of 10 patients who died out of hospital and their family members were uncontactable. This may have led to an underestimation of the true cardiovascular mortality rate.

### 2.4 Laboratory measurements

At the time of hospitalisation, blood tests were taken via venupuncture (fasting not required) and measured for plasma Lp(a) using the particle-enhanced turbidimetric immunoassay with Tina-quant Lipoprotein(a) Gen.2 (Latex) Roche, with inter-assay coefficient of variation (CV) ≤2.2%. The inter-assay CVs for the other lipid particle assays (total cholesterol, high density lipoprotein [HDL-C], directly measured low-density lipoprotein cholesterol [HDL-C] and triglyceride), measured using an enzymatic colorimetry Roche Cobas c702 analyser, were <1.5%.

### 2.5 Statistical analysis

Descriptive statistics of patient demographic and clinical characteristics were reported as number (%) for categorical data, mean ± standard deviation (SD) for normally distributed data, and median and interquartile range (IQR) for non-normally distributed data. Normality of continuous variables was assessed by visual inspection of quantile–quantile (Q–Q) plots. As Lp(a) distributions have a significant positive skew, Lp(a) values were ln-transformed to normalize their distributions for survival analysis. To assess differences in baseline characteristics between the Lp(a) <70 and ≥70nmol/L groups, the Mann–Whitney *U* test was used for continuous characteristics while the chi-squared or Fisher’s exact test was used for categorical characteristics. The study hypothesis was that increased levels of Lp(a) was associated with an increase mortality and MACE risk in patients with IHD admitted to hospital. To detect an all-cause mortality hazard ratio of 2.0 for the Lp(a) ≥70 *vs*. <70nmol/L group with a two-tailed 0.05 significance and 0.80 power, assuming 30% of patients with baseline Lp(a) ≥70nmol/L and an overall 15% probability of all-cause mortality over two years (based on preliminary data), the sample size required using Schoenfeld’s formula was at least 78 events (or estimated total sample size of 519 patients).

Follow-up began on the date of recruitment (during hospital admission) and ended at the earliest of date of cardiovascular event/death or date of last available follow-up. Patients without an event were censored at their last follow-up date. The median and IQR of potential follow-up duration were estimated by the reverse Kaplan–Meier method (i.e. Kaplan–Meier on censored observations treated as events). Adjusted survival curves were generated to illustrate the mortality-free and MACE-free probability over time. The global log-rank test assessed if there were differences in the survival distributions of different groups.

To examine if baseline Lp(a) as a continuous variable and Lp(a) ≥70 nmol/L as a categorical variable were associated with reduced risk of mortality and MACE, we performed univariable Cox proportional hazards regression of time to death and MACE on each explanatory variable. To determine if there was an independent association, we further adjusted for baseline LDL-C, age at first visit, sex, race, body mass index, history of smoking (active smoker or ex-smoker), hypertension, diabetes mellitus, chronic kidney disease, and hospital admission for acute myocardial infarction. All potential confounders and variables of interest were selected *a priori* based on existing literature and expert clinical opinion. We performed simultaneous entry of these variables into the multivariable model, to avoid data-driven variable selection and overfitting (21). Multicollinearity was assessed using the generalized variance inflation factor with values greater than 5 indicating potential multicollinearity. All covariates used in the multivariable regression had values <2 variation inflation factor, indicating minimal collinearity.

The proportional hazards assumption of Cox regression was checked using a test based on Schoenfeld residuals, while the Box-Tidwell test assessed if there was evidence of non-linearity between ln(hazard) and continuous explanatory variables. A p value below 0.05 indicated a violation of the proportional hazards and linearity assumption respectively. Where there was evidence of non-linearity, we used a restricted cubic spline function with three knots at the 10^th^, 50^th^, and 90^th^ percentiles, to model the relationship between continuous explanatory variable and the cardiovascular outcome; this was performed for LDL-C alone. To explore if the association between baseline Lp(a) and cardiovascular outcomes were consistent across age groups, we performed subgroup analysis by age (≤60 and >60 years) at time of hospitalization (8). The unadjusted and adjusted hazard ratios (HRs) with 95% confidence intervals (CIs) were reported.

Discrimination of the multivariable Cox models with versus without Lp(a) was assessed at two years by estimating the time-dependent area under the receiver operating characteristic curve (AUROC), with inverse probability censoring weights to account for right censoring. The Wald test was used to determine if there were differences in the time-dependent AUROCs at two years for all-cause and cardiovascular mortality between the two models. Statistical tests were two-sided with a p<0.05 to be considered for interpretation. All statistical analyses were conducted using Stata 18 (College Station, TX: StataCorp LLC).

## 3 Results

### 3.1 Baseline characteristics

Patient demographic and clinical characteristics are shown in **Table 1**. Overall, median age at admission was 63.5 years (IQR 56.1,71), 82.3% were male, 49.2% were of Chinese descent, and median BMI was 25.0 kg/m^2^ (IQR 22.4,28.4). All participants were admitted to hospital and had a clinical diagnosis of IHD. Half (50%) were admitted for acute myocardial infarction, and the remaining for other reasons including acute decompensated heart failure (13.9%), cardiac symptoms (15.2%), and others (e.g. hypertensive urgency, electrolyte disturbance, arrhythmia, hypotension, cardiac procedure, left ventricular thrombus). Comorbidities present among study participants were hypertension (79%), diabetes mellitus (48.7%), congestive heart failure (33.3%), stroke (12.5%), and chronic kidney disease (24.6%). A high proportion of patients had a history of smoking (51.9%), of whom 29% were active smokers at the time of recruitment. Median baseline Lp(a) was 35.2 nmol/L and 15.8% had hyper-Lp(a) ≥120 nmol/L. Median LDL-C concentration was 2.4 mmol/L; 74.2% had LDL-C ≥1.8 mmol/L and 88.5% had LDL-C ≥1.4 mmol/L. Median serum apolipoprotein B concentration was 0.89 g/L (IQR 0.7, 1.1), while median serum C-reactive protein (CRP) and Troponin T were both elevated at 6.04 mg/L (1.61,20.29) and 95.3 ng/L (19.4, 899), respectively. The use of statin was suboptimal at 68.7% in study participants at baseline.

**Table 1:** Baseline demographic and clinical characteristics of patients admitted to hospital with ischemic heart disease, stratified by baseline lipoprotein(a) [Lp(a)] concentrations <70 nmol/L (normal) with elevated Lp(a) ≥70 nmol/L.

Characteristic	All patients (n=520)	Lp(a) <70 nmol/L (n=369)	Lp(a) ≥70 nmol/L (n=151)	P value
Demographic				
Age (years), median (IQR)	63.5 (56.1,71)	63.6 (56.3,70.6)	62.7 (55.6,72.9)	0.767
Women, n (%)	92 (17.7)	56 (15.2)	36 (23.8)	0.019
Race, n (%)				0.138
Chinese	256 (49.2)	191 (51.8)	65 (43.1)	
Malay	164 (31.5)	111 (30.1)	53 (35.1)	
Indian	66 (12.7)	41 (11.1)	25 (16.6)	
Others	34 (6.5)	26 (7.1)	8 (5.3)	
BMI (kg/m^2^), median (IQR)	25 (22.4,28.4)	25.1 (22.6,28.8)	24.8 (22.2,27.8)	0.209
Comorbidities, n(%)				
Hypertension	411 (79.0)	289 (78.3)	122 (80.8)	0.529
Diabetes mellitus	253 (48.7)	183 (49.6)	70 (46.4)	0.503
Congestive heart failure	173 (33.3)	117 (32.2)	56 (37.6)	0.245
Stroke	65 (12.5)	43 (11.7)	22 (14.8)	0.344
Chronic kidney disease	128 (24.6)	86 (23.3)	42 (27.8)	0.279
History of smoking	270 (51.9)	196 (53.1)	74 (49.0)	0.394
Admitted to hospital for AMI	260 (50)	181 (49.1)	79 (52.3)	0.499
On statin at baseline	357 (68.7)	262 (71)	95 (62.9)	0.071
Blood results, median (IQR)				
LDL-C (mmol/L)	2.40 (1.77,3.46)	2.34 (1.69,3.26)	2.66 (1.96,4.09)	0.004
LDL-C ≥1.8mmol/L, n (%)	386 (74.2)	264 (71.5)	122 (80.8)	0.029
LDL-C ≥1.4mmol/L, n (%)	460 (88.5)	318 (86.2)	142 (94.0)	0.011
HDL-C (mmol/L)	1.13 (0.94,1.34)	1.14 (0.95,1.33)	1.12 (0.93, 1.37)	0.865
Total cholesterol (mmol/L)	4.01 (3.26,5.08)	3.90 (3.19,4.87)	4.22 (3.44,5.45)	0.005
Triglycerides (mmol/L)	1.35 (0.97,2.01)	1.27 (0.95,1.94)	1.51 (1.02,2.17)	0.047
Lp(a) (nmol/L)	35.2 (16.3,80.3)	23.2 (12.1,38.4)	125.8 (89.5,175.5)	–
ApoA1 (g/L)	1.2 (1.05,1.37)	1.21 (1.06,1.37)	1.17 (1.04,1.36)	0.288
ApoB (g/L)	0.89 (0.7,1.1)	0.84 (0.67,1.07)	0.96 (0.81,1.22)	<0.001
CRP (mg/L)	6.04 (1.61,20.29)	6.03 (1.32,20.27)	6.76 (2.19,20.67)	0.316
Troponin T (ng/L)	95.3 (19.4,899)	91 (17.8,929.5)	111 (22.2,825)	0.553
HbA1c (%)	6.4 (5.8,7.8)	6.4 (5.8,7.7)	6.3 (5.7,8.2)	0.938
Creatinine (µmol/L)	94 (76,122)	92 (77,119)	98 (76,129)	0.383
AST (U/L)	26.5 (19,43)	27 (19,44)	25 (18,43)	0.373
ALT (U/L)	23 (17,37)	23 (17,38)	22 (15,35)	0.218

ALT, Alanine transaminase; AMI, Acute myocardial infarction; Apo, Apolipoprotein; AST, Aspartate transaminase; BMI, Body mass index; CRP, C-reactive protein; HbA1c, Hemoglobin A1C; HDL-C, High-density lipoprotein cholesterol; LDL-C, Low-density lipoprotein cholesterol; Lp(a), Lipoprotein(a). History of smoking includes active smoker and ex-smoker; IQR, interquartile range.

There were 151 patients with elevated Lp(a) ≥70nmol/L, comprising 29% of the study cohort, with baseline characteristics described in **Table 1**. The median Lp(a) was 125.8 nmol/L (IQR 89.5,175.5), LDL-C was 2.66 mmol/L, total cholesterol was 4.22 mmol/L, apoB was 0.96 g/L, and CRP was 6.76 mg/L (IQR 2.19, 20.67). Among these patients, 10 did not have standard modifiable risk factors (SMuRF) which were diabetes mellitus, hypertension, and were not active smokers. The median Lp(a) in these 10 patients was 130 nmol/L (range 73.5-208.8 nmol/L). In a subgroup multivariable analysis of patients with elevated Lp(a) ≥70 nmol/L, serum CRP as a continuous variable (natural log transformed) was independently associated with increased risk in all-cause mortality (HR 1.34 [1.01-1.78), *p*=0.041) and cardiovascular mortality (HR 1.52 [1.01-2.27], *p*=0.042), but not MACE.

### 3.2 The impact of lipoprotein(a) on cardiovascular outcomes

The median follow-up duration was 680 days (IQR 576, 746). During the ~2-year follow-up period, 14.6% (76/520), 8.5% (44/520), and 49.2% (256/520) of participants developed all-cause mortality, cardiovascular mortality, and MACE, respectively.

**Table 2** shows the relationship of Lp(a) with cardiovascular outcomes. Using Lp(a) as a continuous variable, univariable analysis revealed that a unit increase in baseline ln(Lp(a)), equivalent to a 2.72-fold increase in Lp(a), was associated with a 25% higher hazard rate of all-cause mortality (HR 1.25, 95% CI [1.02-1.54], *p*=0.034). In multivariable analysis adjusting for baseline LDL-C, age at first visit, sex, race, body mass index, history of smoking, hypertension, diabetes mellitus, chronic kidney disease, and hospital admission for acute myocardial infarction, Lp(a) as a continuous variable was associated with a 26% higher hazard rate of all-cause mortality (HR 1.26 [1.01-1.58], *p*=0.042). However, the relationship between the continuous Lp(a) with cardiovascular mortality and MACE were not statistically significant (HR 1.20 [0.89-1.61], *p*=0.233) and HR 1.02 [0.90-1.14], *p*=0.852 respectively).

**Table 2:** Effect of baseline lipoprotein(a) as a continuous variable (ln[Lp(a)]) and categorical variable (Lp(a) ≥ 70nmol/L *vs <*70 nmol/L) on all-cause mortality, cardiovascular mortality and major adverse cardiovascular event (MACE) in 520 patients at 2 years after hospitalisation.

Outcome and explanatory variable, total no. of events, n (%)	Univariable analysis		Multivariable analysis	
	HR (95% CI)	*p* value	HR (95% CI)	*p* value
All-cause mortality, n=76 (14.6%)				
Lp(a)	1.25 (1.02 - 1.54)	0.034	1.26 (1.01 -1.58)	0.042
Lp(a) ≥ 70nmol/L	1.93 (1.22 - 3.05)	0.005	1.97 (1.20 - 3.22)	0.007
Cardiovascular mortality, n=44 (8.5%)				
Lp(a)	1.24 (0.94 -1.63)	0.126	1.20 (0.89 - 1.61)	0.233
Lp(a) ≥ 70nmol/L	1.93 (1.06 - 3.52)	0.032	2.01 (1.06 - 3.82)	0.033
MACE, n=256 (49.2%)				
Lp(a)	1.08 (0.97 - 1.21)	0.158	1.02 (0.90 - 1.14)	0.852
Lp(a) ≥ 70nmol/L	1.42 (1.09 - 1.84)	0.009	1.29 (0.98 - 1.70)	0.067

Multivariable Cox regression adjusted for baseline LDL-C, age at first visit, sex, race, body mass index, smoker, hypertension, diabetes mellitus, chronic kidney disease, and hospital admission for acute myocardial infarction. Lp(a), lipoprotein(a).

The number of events for each outcome are shown, n(%). Hazard ratio with 95% confidence interval (CI) and *p* value are shown for univariable and multivariable analysis.

**Figure 1:**
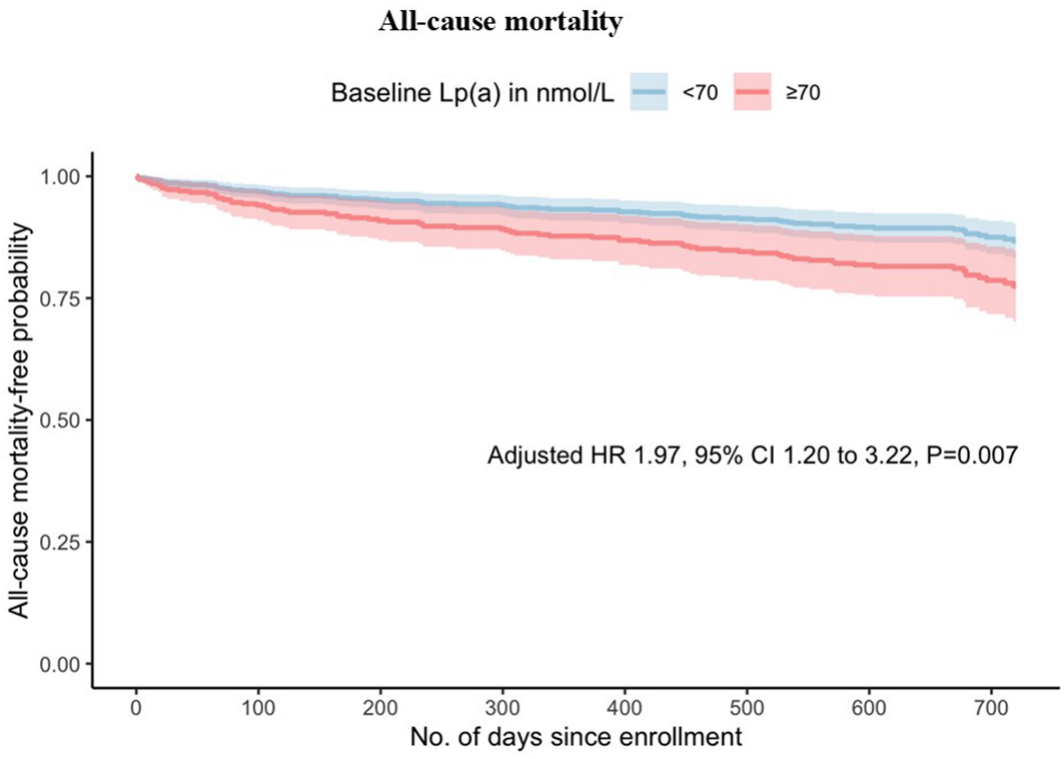
Adjusted survival curves for all-cause mortality against time by baseline lipoprotein(a) above or below 70 nmol/L.

**Figure 2:**
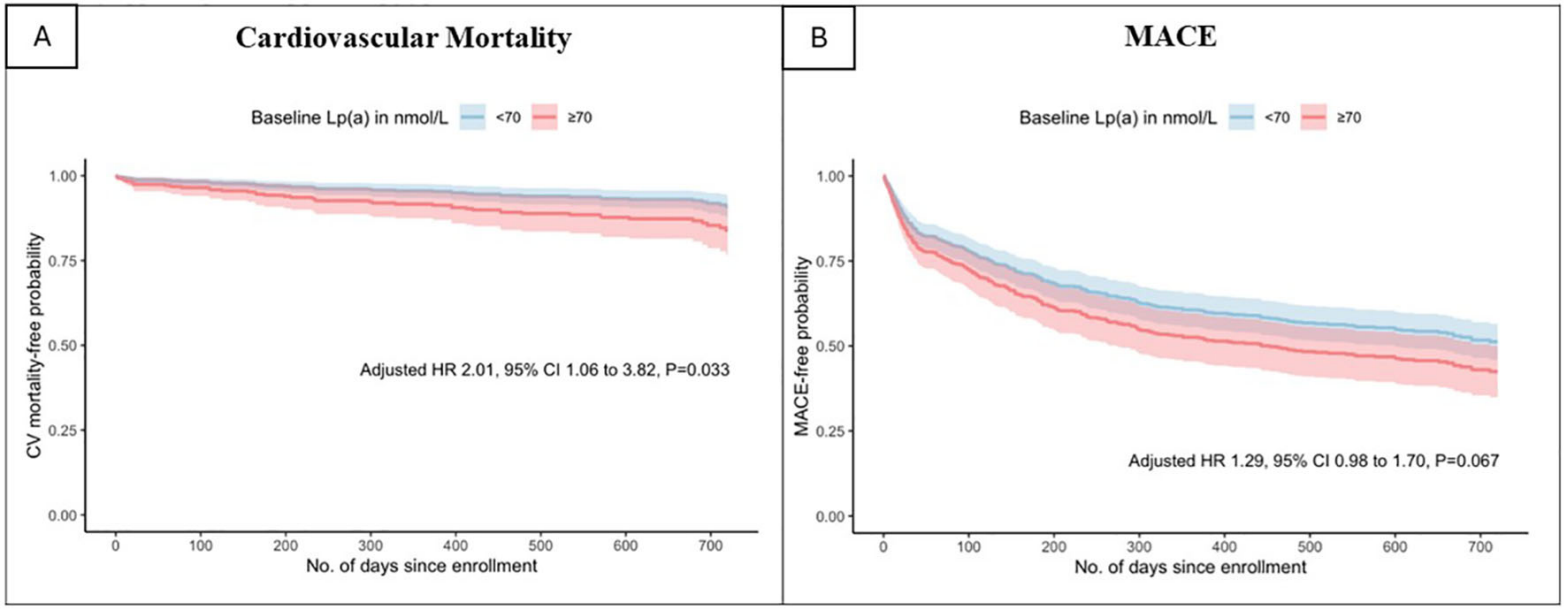
Adjusted survival curves for **(A)** cardiovascular mortality and **(B)**major adverse cardiovascular events by baseline lipoprotein(a) above or below 70 nmol/L.

When analysing elevated Lp(a) as a categorical variable in univariable analysis, Lp(a) ≥70nmol/L was found to be a predictor of all-cause mortality (HR 1.93 [1.22-3.05], *p*=0.005), cardiovascular mortality (HR 1.93 [1.06-3.52], *p*=0.032) and MACE (HR 1.42 [1.09-1.84], *p*=0.009). In the multivariable analysis, Lp(a) ≥70 nmol/L remained significantly associated with increased risk of all-cause mortality (HR 1.97 [1.20-3.22], *p*=0.007) and cardiovascular mortality (HR 2.01 [1.06-3.82], *p* =0.033) but not MACE (HR 1.29 [0.98-1.70], *p*=0.067). As shown in **Figures 1** and **2**, the adjusted survival curves (and similarly for Kaplan Meier curves, not shown) showed an increased incidence of all-cause mortality and cardiovascular mortality in patients with elevated Lp(a) ≥70nmol/L compared to those with Lp(a) <70 nmol/L.

**Figure 3:**
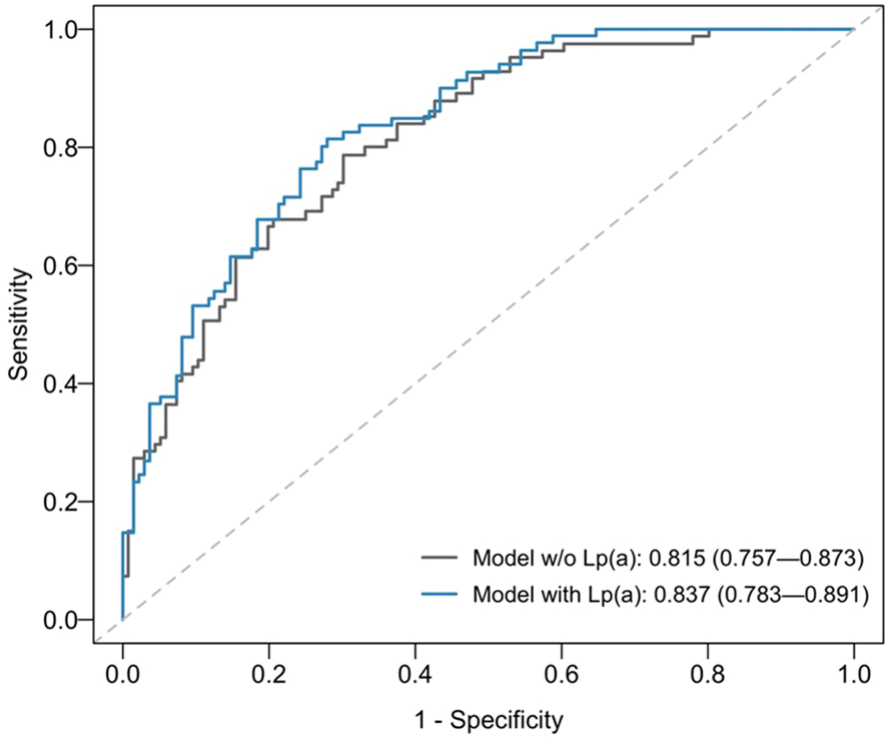
Time-dependent area-under-receiving operating curves (AUROCs) at two years for all-cause mortality of multivariable model without (w/o) Lp(a) [grey line] versus model with baseline Lp(a) < 70 nmol/L compared with ≥70nmol/L [blue line].The multivariable Cox proportional hazards regression models included baseline LDL-C, age at first visit, sex, race, body mass index, smoker, hypertension, diabetes mellitus, chronic kidney disease, and hospital admission for acute myocardial infarction as covariates.

When stratified by age, 36.9% (192/520) of patients were ≤60 years at time of hospitalisation, while 63.1% (328/520) were >60 years. Subgroup analysis by age ≤60 compared with age >60 years at hospitalisation showed that Lp(a) and Lp(a)≥70 nmol/L remained statistically significant with increased all-cause mortality for subgroup of age>60 but not for the subgroup of age ≤60 years (**Table 3**). The time-dependent AUROC at two years for all-cause mortality was higher in the multivariable model with baseline Lp(a) at 0.837 [95% CI 0.783 to 0.891], compared to 0.815 [95% CI 0.757 to 0.873] in the model without Lp(a) (P=0.045; **Figure 3**). Consistent results were obtained for cardiovascular mortality. The model with Lp(a) had a time-dependent AUROC at two years of 0.844 [95% CI 0.785 to 0.903], compared to 0.818 [95% CI 0.752 to 0.884] in the model without Lp(a) (P=0.064).

**Table 3:** Subgroup analysis of Lp(a) with all-cause mortality, cardiovascular mortality and MACE, stratified by age (>60 years and ≤60 years) at inpatient hospitalisation.

Subgroup, outcome, and explanatory variable, total no. of events (%)	Adjusted* HR (95% CI)	P value
≤60 years at admission		
All-cause mortality, n=13 (6.8%)		
Lp(a)	1.49 (0.83 to 2.68)	0.182
Lp(a) ≥70nmol/L (*vs*. <70)	3.81 (0.94 to 15.46)	0.061
Cardiovascular mortality, n=6 (3.1%)		
Lp(a)	1.69 (0.72 to 3.96)	0.231
Lp(a) ≥70nmol/L (*vs*. <70)	†	†
MACE, n=85 (44.3%)		
Lp(a)	1.22 (0.99 to 1.50)	0.063
Lp(a) ≥70nmol/L (*vs*. <70)	1.53 (0.95 to 2.47)	0.077
>60 years at admission		
All-cause mortality, n=63 (19.2%)		
Lp(a)	1.28 (1.00 to 1.65)	0.049
Lp(a) ≥70nmol/L (*vs*. <70)	1.98 (1.14 to 3.43)	0.015
Cardiovascular mortality, n=38 (11.6%)		
Lp(a)	1.25 (0.90 to 1.73)	0.176
Lp(a) ≥70nmol/L (*vs*. <70)	1.88 (0.93 to 3.81)	0.080
MACE, n=171 (52.1%)		
Lp(a)	0.94 (0.80 to 1.09)	0.410
Lp(a) ≥70nmol/L (*vs*. <70)	1.21 (0.86 to 1.69)	0.282

*All multivariable Cox regression models adjusted for baseline LDL-C, age at first visit, sex, race, body mass index, smoker, hypertension, diabetes mellitus, chronic kidney disease, and hospital admission for acute myocardial infarction.

†Excluded as model coefficients were unstable with only six cardiovascular mortality events in this subgroup.

CI, Confidence interval; HR, Hazard ratio; Lp(a), Lipoprotein(a); MACE, Major adverse cardiovascular events.

Baseline lipoprotein(a) expressed as continuous variable (ln[Lp(a)]) and categorical variable (Lp(a) ≥ 70nmol/L *vs <*70 nmol/L).

## 4 Discussion

Our multi-ethnic prospective observational study uniquely reports that Lp(a) is a positive predictor of increased mortality risk when tested in patients with IHD admitted to hospital. In this very high-risk group, we found that elevated Lp(a), as a continuous variable or using a risk threshold ≥ 70 nmol/L was a strong independent predictor of mortality two years post-hospitalization. Our findings add significantly to current literature and reinforces international recommendations to identify patients with high Lp(a), particularly those already at very high risk of ASCVD to enhance strategies for reducing overall cardiovascular risk (1). Our study findings also add to previous efforts to understand the relationship between Lp(a) and cardiovascular disease in multi-ethnic Asians patients.

We previously reported that hyper-Lp(a) ≥120 nmol/L was an independent risk factor of CAD and associated with CAD severity in a study of 2,025 patients that underwent coronary angiography (22). In that study, Lp(a) ≥130 nmol/L was associated with increased risk of AMI (odds ratio (OR) 1.47 [1.02-2.10], *p*=0.038) and Lp(a) ≥160 nmol/L was associated with 2 times increased risk of AMI (OR 1.96 [1.20-3.19], *p*=0.007) (22). Interestingly, our current study identifies a lower risk threshold of Lp(a) ≥70 nmol/L as a predictor of mortality two years after hospitalization, suggesting that even mildly elevated Lp(a) is a significant risk factor in patients at the highest cardiovascular risk. This may reflect the characteristics of our high-risk cohort, including hospitalization for cardiovascular events, Asian ethnicity, a high prevalence of prior myocardial infarction, and significant comorbidities such as active smoking (29%), history of smoking (52%), diabetes (48.7%) and hypertension (79%). From the subgroup analysis, we found that Lp(a) was associated with increased risk of all-cause mortality for the subgroup age > 60 years and not statistically significant for those with age ≤ 60 years (borderline *p* value with Lp(a) ≥ 70 nmol/L). Interestingly, a recent analysis of the NHANES data reported that higher Lp(a) quartile compared with lower Lp(a) quartile was associated with all-cause and cardiovascular mortality only in age >60 and not age ≤ 60 years (8). There are multiple thresholds of abnormal Lp(a) in medical literature and consensus statements which includes 30 mg/dL, 32 nmol/L, 45 nmol/L, 75 nmol/L and 70 nmol/L (1, 2, 23, 24). The 2022 European consensus defined abnormal Lp(a) > 75 nmol/L while the 2024 NLA scientific statement defined Lp(a) ≥75 nmol/L to be abnormal (1, 24). Regardless of threshold used, the cardiovascular risk and mortality risk is linearly associated with increasing Lp(a) concentrations (7). When the Lp(a) ≥75 nmol/L was used as threshold for analysis in this paper, our results remained similar in hazard ratios.

Extrapolating from genetic analyses, a single Lp(a) particle is estimated to be 6 times more atherogenic than a single LDL particle, despite both types of lipid particle having a single apoB particle (25). The atherogenic mechanisms of Lp(a) are attributed to the proatherogenic effects of apolipoprotein(a), the proinflammatory effects of oxidised phospholipids carried within Lp(a), and the inhibition of plasminogen activity, which promotes thrombosis (3). Lp(a) levels are mostly genetically determined with adult blood Lp(a) concentrations reached at age of 5 years old for most people (4), with 10-20% variability as age increases (26). Therefore, the chronic and perhaps early exposure of Lp(a) in high blood concentrations increases risk of myocardial infarction, premature onset of IHD and cardiovascular mortality. Apart from these conditions, elevated Lp(a) concentrations are risk factors for calcific aortic valve stenosis, abdominal aortic aneurysms, peripheral artery disease, and major adverse limb events (27). Thus, the importance of Lp(a) as a cardiovascular risk factor and predictor should not be overlooked and needs to be integrated into clinical pathways to support intensification of modifiable risk factor management.

Put together, our findings support the conclusion that the cardiovascular risks conferred to individuals by Lp(a) widely vary according to 2 major factors; firstly, the Lp(a) concentration and secondly, the patient’s baseline cardiovascular risk (1). Supporting the first point, the analysis of 460,506 people in the UK Biobank by Patel et al. showed that a linear relationship of Lp(a) concentrations and risk of ASCVD exists with an inflection point at approximately 30–50 nmol/l of Lp(a) concentration (7). Similarly, a large retrospective analysis of 16,419 individuals in Boston by Berman et al. with a median follow-up of 11.9 years, demonstrated a linear relationship between Lp(a) concentration and the risk of future MACE events in the primary prevention cohort (28). The latter study reported that every category of Lp(a) percentile was associated with much higher (at least double) future incident rates of MACE events in patients with ASCVD, compared to those without ASCVD at baseline (28).

However, the interpretation of Lp(a)-induced risk in patients with ASCVD is less straightforward (7, 28); In the UK Biobank analysis, Lp(a) ≥150 nmol/L was associated with higher relative risk for future ASCVD (median follow-up of 11.2 years) in individuals without ASCVD at study baseline, compared to individuals with ASCVD at baseline (HR 1.50 [1.44-1.56] *vs* HR 1.16 [1.06-1.27]). In the USA retrospective study by Berman et al, there was a plateau in ASCVD risk conferred by Lp(a) in the secondary prevention cohort when Lp(a) was above the 70^th^ percentile, unlike the primary prevention cohort (28). The authors of both studies postulated that this is explained by the aggressive use of statin or other preventive therapy in patients with ASCVD, which may have lowered Lp(a)-mediated risk estimation (7, 28). We concur that this may also potentially explain the inconsistent reports of studies investigating the prognostic value of Lp(a) in patients with ACS, with some showing that Lp(a) is not associated with MACE or all-cause mortality (11–13, 29). On the contrary, pooling together data from all published studies, a recent meta-analysis of 18,168 patients reported that elevated Lp(a) is a risk factor that increased risk of MACE (HR 1.26 [1.17–1.35], *p*<0.001) and all-cause mortality (HR 1.36 [1.05–1.76], *p*=0.02) in patients with ACS (11). In the subgroup analyses of this meta-analysis, the authors mentioned that Lp(a) >30 mg/dL (≈ 70nmol/L) threshold compared with Lp(a) ≤30 mg/dL as well as comparing Europe *vs* Asia studies, were associated with an increased risk of MACE but not statistically significant for all-cause mortality, but results were not shown and unclear whether the association of Lp(a) and MACE was higher in European or Asian studies (11). However, the heterogeneity of the study designs, follow-up duration, measurement methods and the diverse threshold values for elevated Lp(a) across the studies could affect the results particularly sub-analyses of this meta-analysis (11). More studies investigating the lower threshold of Lp(a) >70 nmol/L in patients with IHD or ACS and of various ethnicities would be insightful.

The main limitation of our study is the modest sample size, which may explain the lack of statistically significant findings for MACE. We could not perform sub-analysis of groups of interest, by ethnicity, gender or categorisations of Lp(a) at higher concentrations. Lp(a) levels may be falsely decreased or increased during acute events (e.g. myocardial infarction and sepsis), and a repeat Lp(a) at outpatient setting is recommended (30, 31). In this study, we did not have sufficient repeat Lp(a) levels of all patients to correlate variability of Lp(a) over time with clinical outcomes. Another limitation was that our study data is not generalizable to patients with IHD that never required hospital admissions. Sub-analysis requires cautious interpretation because of low statistical power. However, the major strength of our study is being the first Asian report, to demonstrate the significance of Lp(a) in patients hospitalised with IHD, i.e. a very high-risk patient group, using the uniform measurement using a relatively isoform-insensitive assays that quantifies Lp(a) in molar rather than mass concentrations.

In conclusion, our findings show that elevated Lp(a) is an independent risk factor associated with an increased risk for all-cause mortality and cardiovascular mortality in patients hospitalised with IHD. This supports the expert recommendations that all patients at high risk for ASCVD should have their Lp(a) tested at least once in their adult lifetime (1–3). Although the prevalence of elevated Lp(a) is lower among Asians than in Caucasians and Blacks (7), our previous work and this study put together showed that elevated Lp(a) is indeed an important cardiovascular risk factor among Asians; Elevated Lp(a) is a predictor of IHD onset and severity (22), as well as cardiovascular and all-cause mortality. Thus, our study findings support the call by multiple consensus statement and guideline for more awareness and training among healthcare professionals to manage Lp(a) (32), and importantly, integration of Lp(a) as a compulsory testing in all adult Asians at high cardiovascular risk. Detection of elevated Lp(a) will allow for important mitigation strategies such as lower LDL-C attainment targets and other risk factor management strategies (1–3). However, gaps in clinical practice related to Lp(a) such as standardisation of assays, reporting units and cost-effectiveness still needs to be addressed.

## Data Availability

The original contributions presented in the study are included in the article/**Supplementary Material**. Further inquiries can be directed to the corresponding author.

## Supplementary material

Supplementary Figure 1 A CONSORT flow chart of patients recruited and consented to this observational study.
