# Supplementary figures and images for: Lipoprotein(a) as a predictor of mortality in hospitalised patients with ischaemic heart disease
Source: Front Endocrinol (Lausanne). 2025 Jul 22;16:1541712. doi: 10.3389/fendo.2025.1541712 (PMC12321530; doi:10.3389/fendo.2025.1541712)

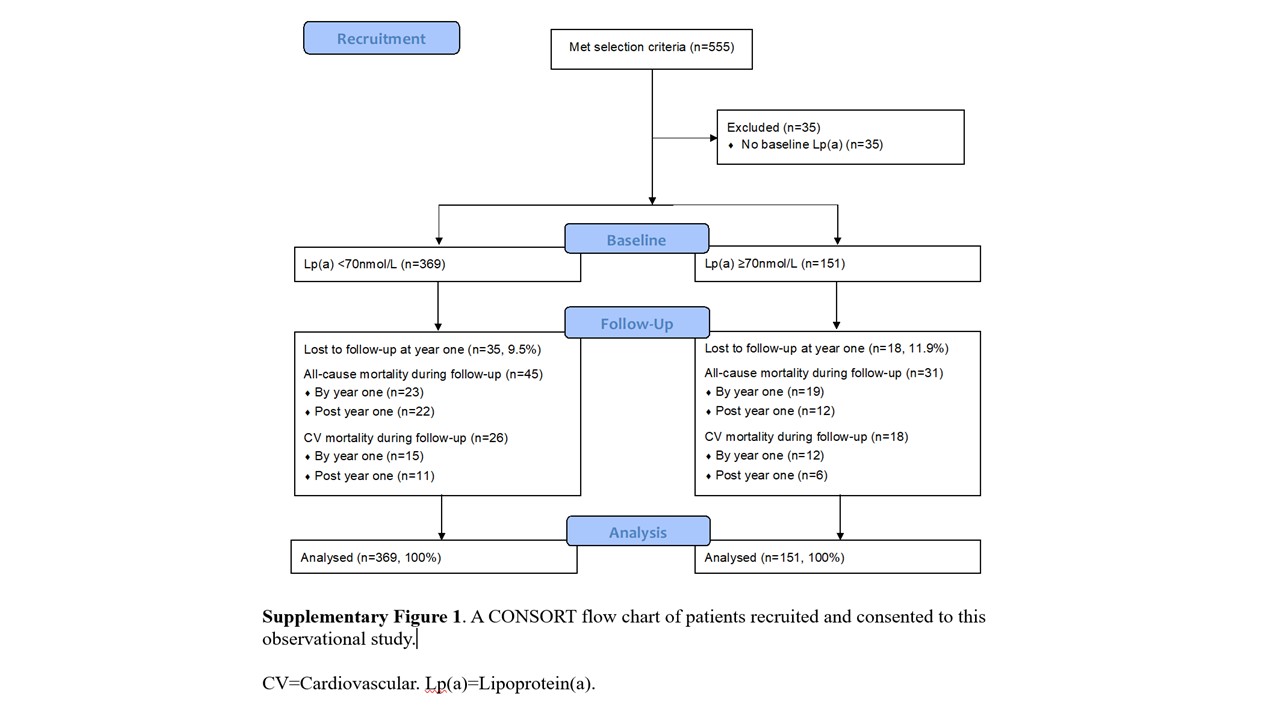

Supplement: Supplementary Figure 1 — A CONSORT flow chart of patients recruited and consented to this observational study. [file Image1.jpeg]
